# Supplementary material for: Design, synthesis, and in vitro evaluation of a carbamazepine derivative with antitumor potential in a model of Acute Lymphoblastic Leukemia
Source: PLoS One. 2025 Apr 28;20(4):e0319415. doi: 10.1371/journal.pone.0319415 (PMC12036894; doi:10.1371/journal.pone.0319415)

**SUPPORTING INFORMATION**

**Design, Synthesis, and In Vitro Evaluation of a Carbamazepine Derivative with Antitumor Potential in a Model of Acute Lymphoblastic Leukemia**

Cristian Álvarez-Gómez, Angela V. Fonseca-Benítez, James Guevara-Pulido

^1^INQA, Química Farmacéutica, Universidad El Bosque, Bogotá, Colombia

Corresponding author joguevara@unbosque.edu.co

# S1 Fig. Pearson Correlation Descriptor Vs Descriptor


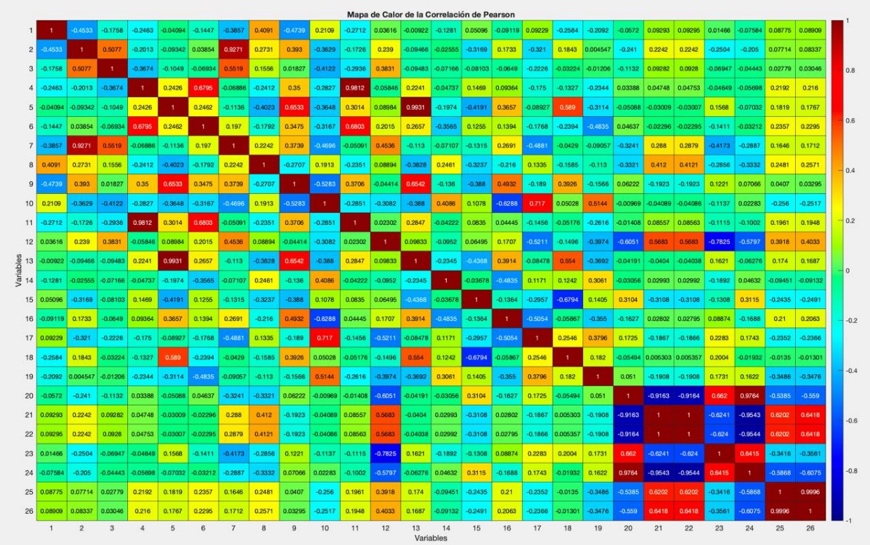


# S2 Fig. Pearson Correlation Descriptor Vs. IC_50_


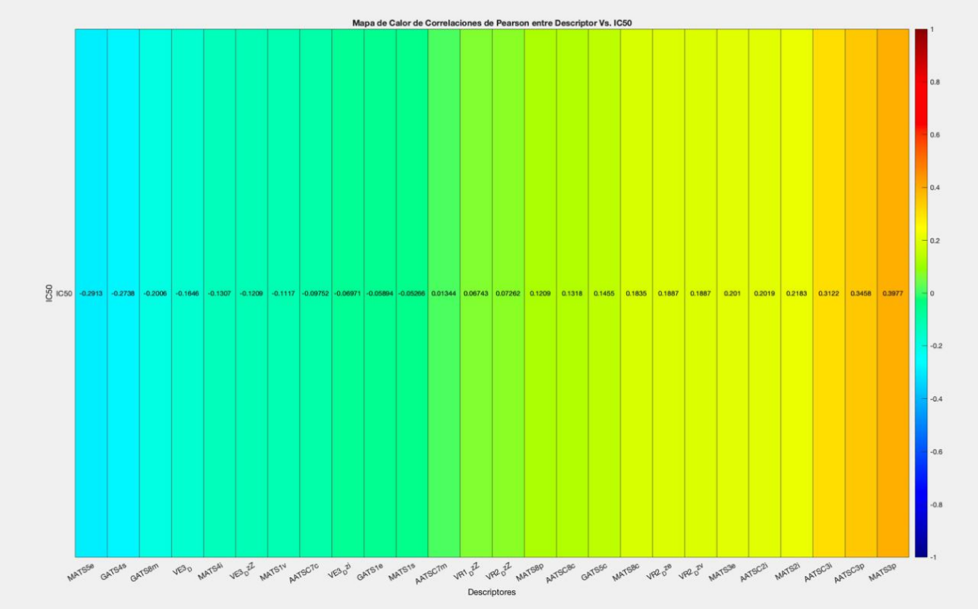

Supplement: S1 and S2 Fig — (DOCX) [file pone.0319415.s001.docx]
