# Supplementary material for: Design, synthesis, and in vitro evaluation of a carbamazepine derivative with antitumor potential in a model of Acute Lymphoblastic Leukemia
Source: PLoS One. 2025 Apr 28;20(4):e0319415. doi: 10.1371/journal.pone.0319415 (PMC12036894; doi:10.1371/journal.pone.0319415)
Supplement: S2 File — (DOCX) [file pone.0319415.s002.docx]

**SUPPORTING INFORMATION**

**Design, Synthesis, and In Vitro Evaluation of a Carbamazepine Derivative with Antitumor Potential in a Model of Acute Lymphoblastic Leukemia**

Cristian Álvarez-Gómez, Angela V. Fonseca-Benítez, James Guevara-Pulido

^1^INQA, Química Farmacéutica, Universidad El Bosque, Bogotá, Colombia

Corresponding author joguevara@unbosque.edu.co

# NMR-H^1^ **CR80**


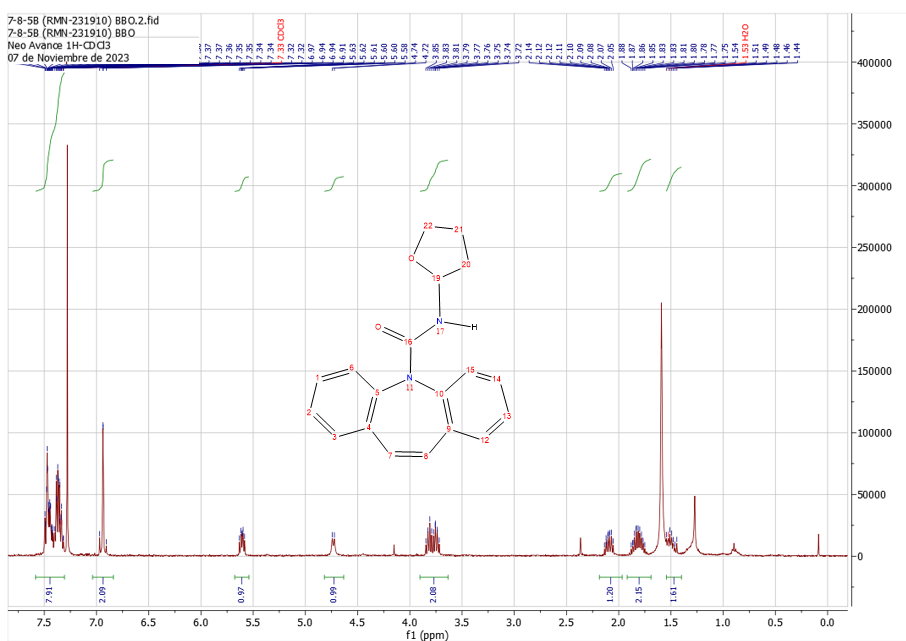


# NMR-C^13^ **CR80**


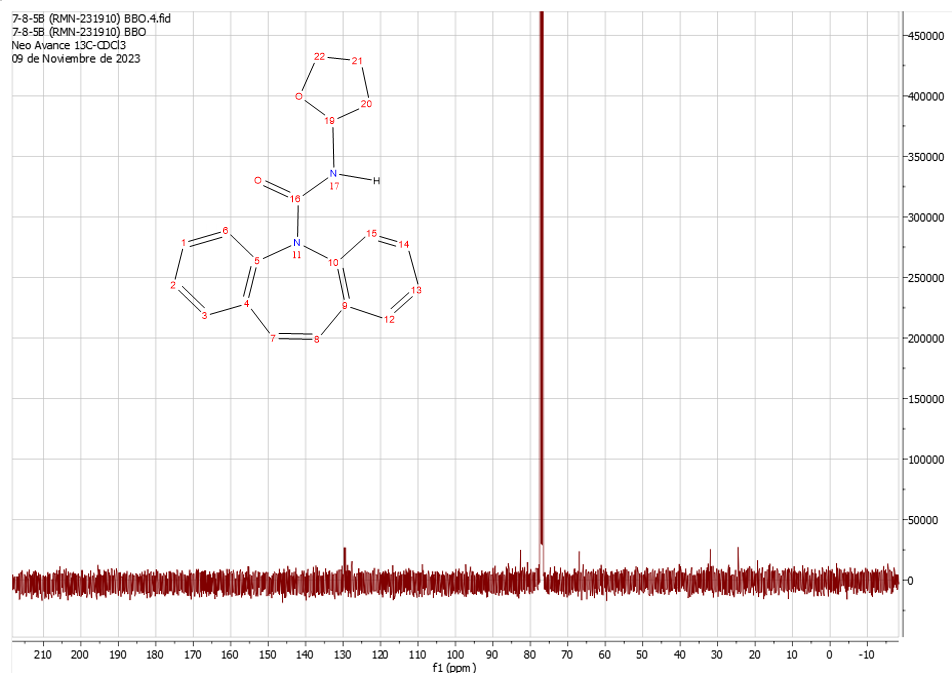


# COSY **CR80**


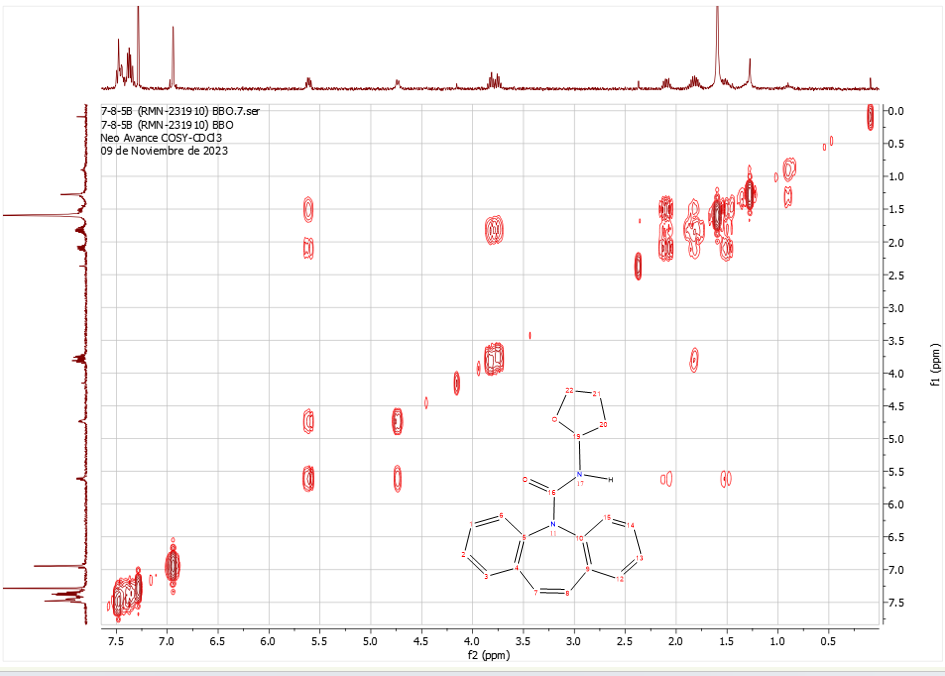


# HSQC **CR80**


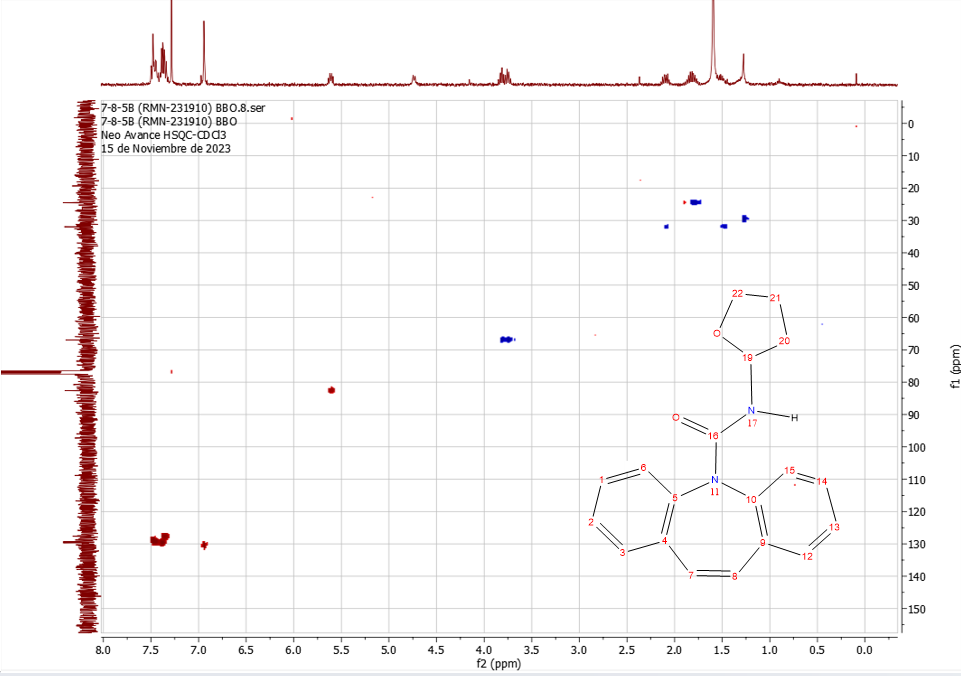


# DRX


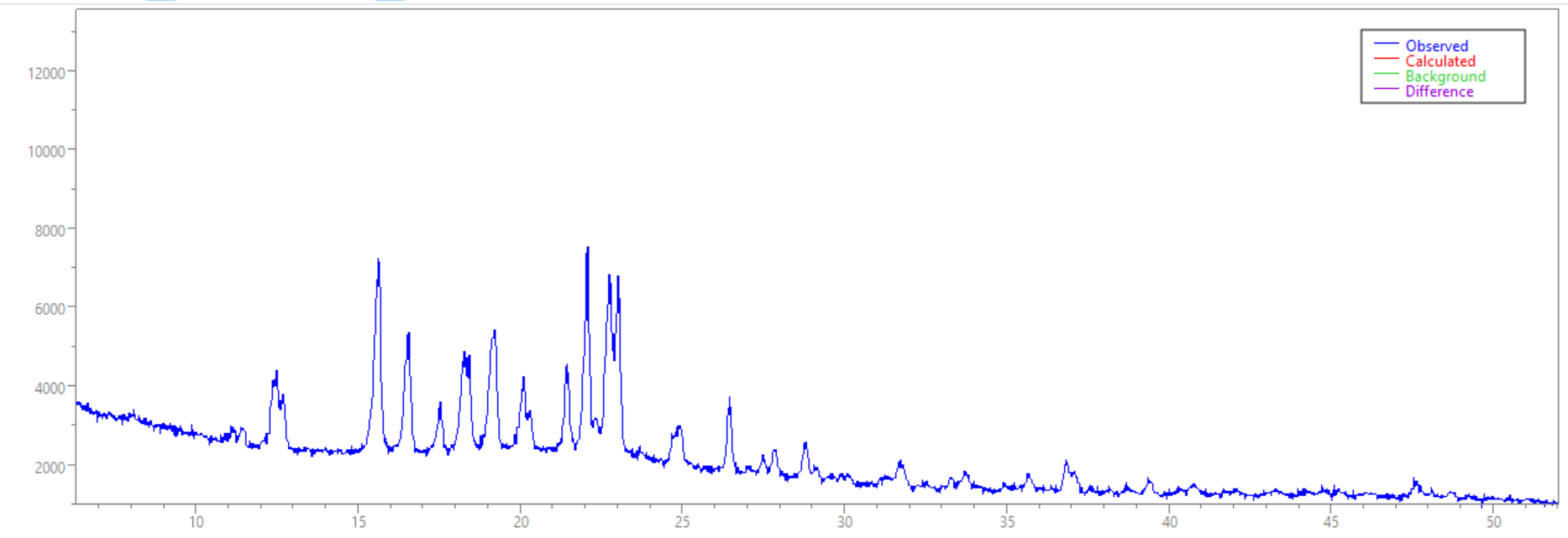


Starting from the 1485.48Ȧ3, with quality parameters adjusted in the determination of the network parameters, M( 20) = 18.1 and F( 31) = 16.4(0.0119, 158) as shown below, with the cell constants Through a whole powder pattern decomposition (WPPD) analysis and systematic absences, a spatial group of P1211 was determined, which was corroborated with the WPPD with the adjustment of their maximum XRD from 10° to up to 40°2θ


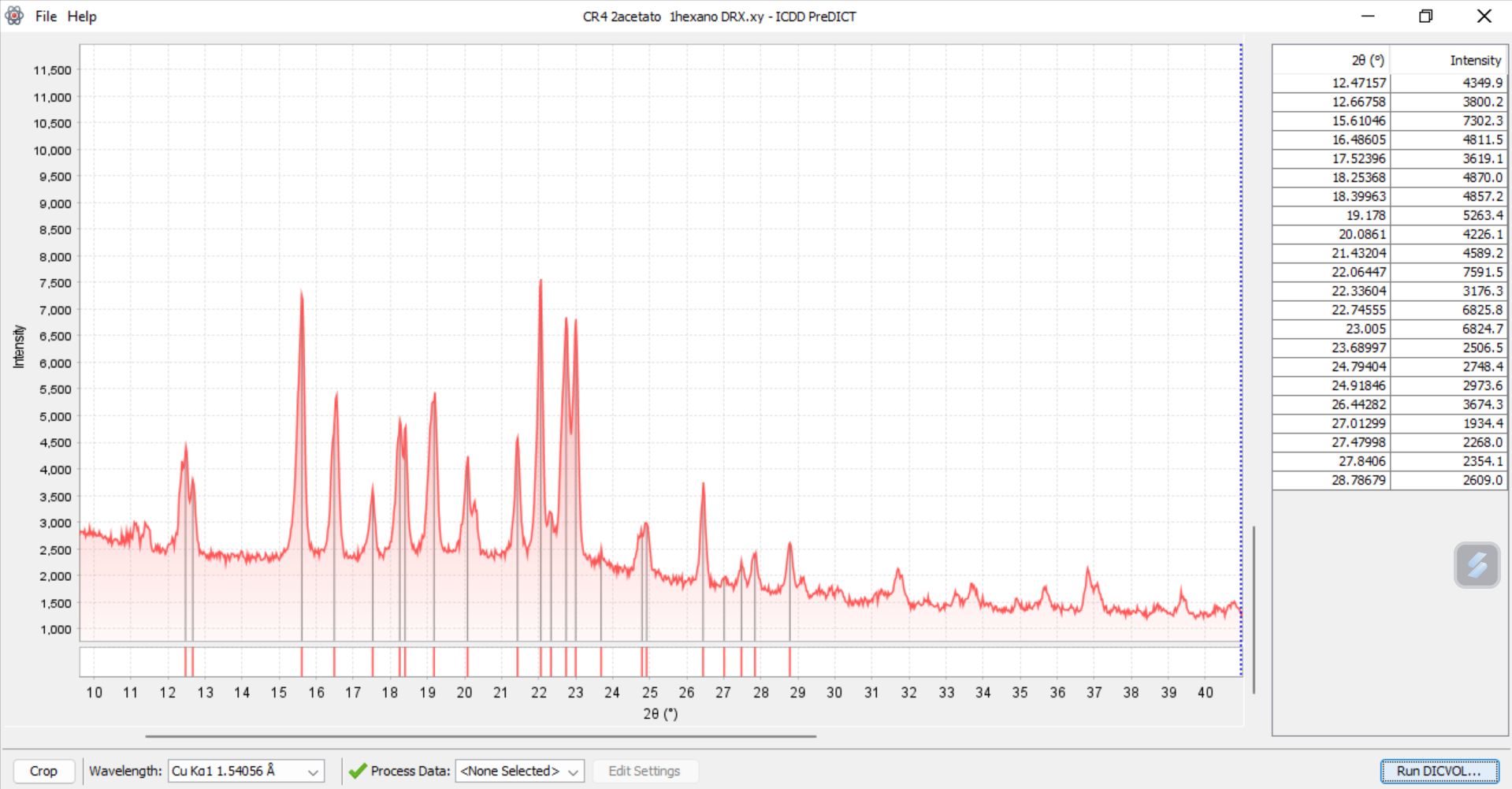


**IR**


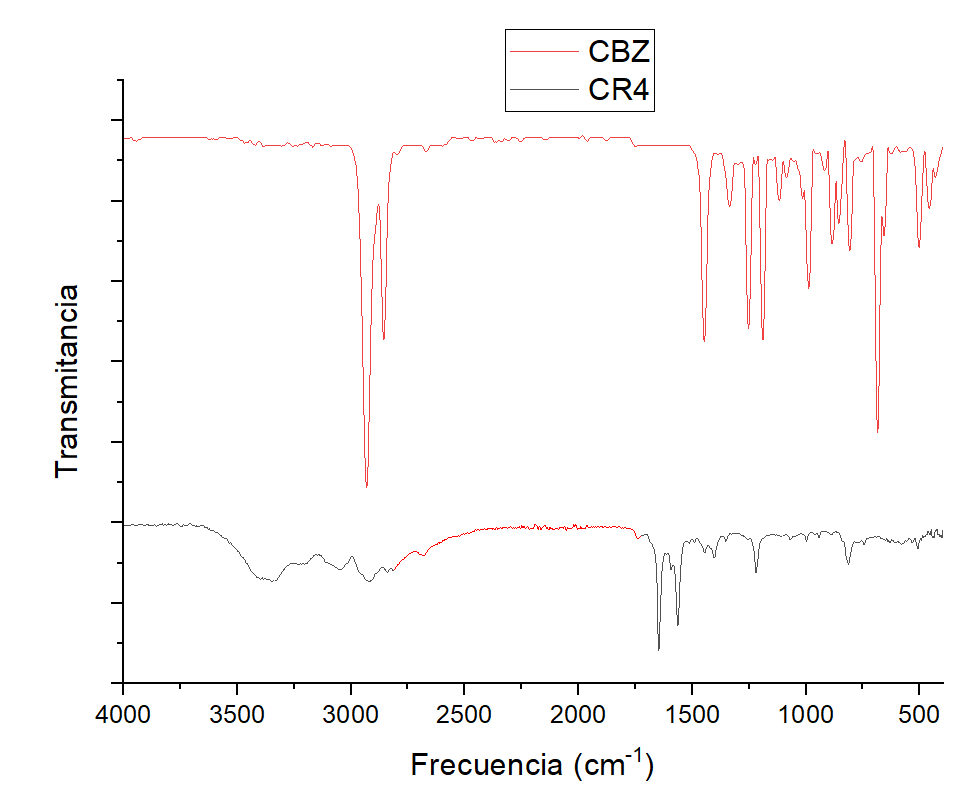


MS-HR **CR80** CHARACTERIZATION
