# Supplementary material for: Design, synthesis, and in vitro evaluation of a carbamazepine derivative with antitumor potential in a model of Acute Lymphoblastic Leukemia
Source: PLoS One. 2025 Apr 28;20(4):e0319415. doi: 10.1371/journal.pone.0319415 (PMC12036894; doi:10.1371/journal.pone.0319415)
Supplement: S3 Table — (DOCX) [file pone.0319415.s003.docx]

**SUPPORTING INFORMATION**

**Design, Synthesis, and In Vitro Evaluation of a Carbamazepine Derivative with Antitumor Potential in a Model of Acute Lymphoblastic Leukemia**

Cristian Álvarez-Gómez, Angela V. Fonseca-Benítez, James Guevara-Pulido

^1^INQA, Química Farmacéutica, Universidad El Bosque, Bogotá, Colombia

Corresponding author joguevara@unbosque.edu.co

# **Table 1SI**. Molecule structures used for INQA-ANN training

|   **1** |   **2** |   **3** |   **4** |   **5** |   **6** |
| --- | --- | --- | --- | --- | --- |
|   **7** |   **8** |   **9** |   **10** |   **11** |   **12** |
|   **13** |   **14** |   **15** |   **16** |   **17** |   **18** |
|   **19** | |   **20** | |   **21** | |

# **Table 2SI.** Molecular descriptors used for training the QSAR model

|  | Descriptor | Descriptor description |
| --- | --- | --- |
| 1 | AATSC7c | Broto-Moreau autocorrelation - lag 7 / weighed by loads |
| 2 | AATSC7m | Broto-Moreau autocorrelation - lag 7 / weighed by mass |
| 3 | AATSC3p | Broto-Moreau autocorrelation - lag 3 / heavy due to polarizabilities |
| 4 | AATSC2i | Broto-Moreau autocorrelation - lag 2 / heavy by ionizables |
| 5 | MATS8c | Moran autocorrelation - lag 8 / weighed by loads |
| 6 | MATS3e | Moran autocorrelation - lag 8 / heavy by Sanderson electronegativities |
