# Supplementary material for: Design, synthesis, and in vitro evaluation of a carbamazepine derivative with antitumor potential in a model of Acute Lymphoblastic Leukemia
Source: PLoS One. 2025 Apr 28;20(4):e0319415. doi: 10.1371/journal.pone.0319415 (PMC12036894; doi:10.1371/journal.pone.0319415)
Supplement: Attachment — Submitted filename: Response Reviewers .docx [file pone.0319415.s007.docx]

**Sapan Kamleshkumar Shah, Ph.D., M.Pharm.**

**Academic Editor**

**PLOS ONE**

We have addressed each of the comments provided by the academic editor. The referees and the PlosOne technical team appreciate the level of detail that results in the quality of the manuscript. Below, we present the adjustments made to the documents and the explanations that gave rise.

**Journal requirements:**

When submitting your revision, we need you to address these additional requirements.

Please ensure that your manuscript meets PLOS ONE's style requirements, including those for file naming.

- **Response:** The manuscript was corrected in its entirety according to the requirements of the journal

Thank you for stating the following in the Acknowledgments Section of your manuscript:

“This project was funded by the INQA Research Group, which is associated with the Pharmaceutical Chemistry Program at Universidad El Bosque, Bogotá, Colombia.”

We note that you have provided funding information that is not currently declared in your Funding Statement. However, funding information should not appear in the Acknowledgments section or other areas of your manuscript. We will only publish funding information present in the Funding Statement section of the online submission form. Please remove any funding-related text from the manuscript and let us know how you would like to update your Funding Statement.

- **Response:** Thanks for the clarification; the statement was withdrawn, and the financing does not need to be added again in another section.

Please include captions for your Supporting Information files at the end of your manuscript, and update any in-text citations to match accordingly. Please see our Supporting Information guidelines for more information: <http://journals.plos.org/plosone/s/supporting-information>.

**• Response:** The information was added and corrected according to all the magazine's criteria

**Additional Editor Comments:**

Abstract need to be refurbish. Many grammatical mistakes are provided in abstract.

- **Response:** Thanks for the correction. All grammatical errors were corrected.

Acute lymphoblastic leukemia (ALL) is a significant concern in both pediatric and adult demographics. Despite 156 approved cancer therapies based on small molecules, a mere five apply to all types of leukemia. Unfortunately, adherence to these treatments is low due to adverse side effects. Consequently, there is an urgent need to identify more effective treatment options for ALL. This study presents a potential solution. We have designed over fifty analogs of carbamazepine, utilizing a combination of ligand-based and structure-based drug design methodologies. Among these analogs, we identified the CR80 analog, which demonstrated predicted binding values of -8.66 kcal/mol against beta-tubulin, a favorable LogP, and IC50 values suitable for in vitro evaluation. The CR80 compound was synthesized with a yield of 50% and subsequently assessed in vitro against the U-937 cell line. It obtained an IC50 value of 800 nM to 1000 nM and a selectivity index of two, thus marking it as a promising candidate for in vivo studies.

At least 5 key words should be added by authors.

- **Response:** Beta-tubulin, drug discovery, and in vitro were added as keywords

The statement “The affinity of ALL and beta-tubulin drugs and their designed analogs was evaluated in kcal/mol using the PDB 6QUS crystal structure” not justified?

- **Response:** thank you for the comment. Indeed, we had not explained this statement in the best way. It was corrected as follows.

“The affinity of drugs with pharmacological activity against beta-tubulin, their designed analogs, and other drugs commonly used for the treatment of ALL was evaluated in kcal/mol using the PDB 6QUS crystal structure.

Sentence never start with value eg. “10 mg of CR80 was added to 10 mL of (PBS, 1X pH 7.35)” grammatically incorrect.

- **Response:** Thank you for the correction.

“Ten milligrams of CR80 were added to ten milliliters of phosphate-buffered saline (PBS) at a concentration of 1X and a pH of 7.35.”

What is meaning of this “CR80 dissolved in Ethyl Acetate 451.2;121.2; y 41.01 µM.??”

- **Response**: We wanted to say that the concentration of CR80 in PBS was established by interpolating the calibration curve of CR80, which exhibits a concentration range of 41 to 451 micromolar. This was corrected in the manuscript.

Overall, authors utilized only 21 molecules for QSAR model building. These no. is too less for building statistically robust QSAR. Further authors have not validated QSAR model according to OECD guidelines? So, applicability of build model for screening external dataset is doubtful and meaningless as authors have not even performed splitting of dataset, internal validation and Applicability domain studies?

- **Response:** In agreement with the comments, we allow ourselves to give the following explanation. In the most recent review published in July 2024 [1] the authors describe how a small number of data with a correct choice can offer a robust QSAR model “Despite the need for large training datasets being the 'Achilles' heel' of deep learning in drug discovery, several advances allow neural networks to be – paradoxically – powerful tools in low-data scenarios. An increasing body of literature shows how strategies like the ones discussed in this minireview can lead to high-performing deep learning models, even with little data.” In our QSAR model we reduce the noise of the model by carrying out an in-depth bibliographic review and grouping molecules with similar electronic and steric properties with a data normalization that allows a robust model verified by the internal validation of the model R2=0.734 and an external validation that exceeds the parameters described in the literature as described in graph 1.

For greater clarity, the following paragraph has been added in the document.

The results are displayed in graph 1a, showing an R² = 0.734 and including the model's cross-validation, demonstrating the validity of the built QSAR model. With a small amount of data but a strict selection, the model's noise is reduced, obtaining a model with high predictive capacity [2].

[1]Tilborg, D., Brinkmann, H., Criscuolo, E., Rossen, L., Özçelik, R., & Grisoni, F. (2024). Deep learning for low-data drug discovery: hurdles and opportunities. *Current Opinion in Structural Biology*, *86*, 102818.

[2] Golbraikh, A., & Tropsha, A. (2000). Predictive QSAR modeling based on diversity sampling of experimental datasets for the training and test set selection. *Molecular diversity*, *5*, 231-243.

Authors have performed molecular docking studies on selected 21 molecules. However, they have not performed any cross validation studies of docking results even with standard inhibitors of beta tubulin or native inhibitors of 6QUS taxol?

- **Response:** According to your question the docking was validated by docking with the ligand with activity demonstrated by beta tubulin vincristine [3] the energy was evaluated, and based on it the comparison was made as described in table 1.

The document was clarified for readers.

[3] Reichle, A., Diddens, H., Altmayr, F., Rastetter, J., & Andreesen, R. (1995). Beta-tubulin and P-glycoprotein: major determinants of vincristine accumulation in B-CLL cells. *Leukemia research*, *19*(11), 823-829.

Authors used both approach LBDD and SBDD but not correlated both approached design?

- **Response**. According to your question, we would like to clarify that of course, we correlate both models; the selection criteria for our candidates are the following as described in the manuscript,

1. Affinity by Docking
2. IC50 By QSAR model
3. ADME evaluation

We additionally predict the IC_50_ values and affinities for the molecular target and calculate the logP and toxicity values of the hit molecules using admelab 3.0 [26]. This helps us establish minimum quality criteria and design molecules that improve all pharmacological attributes evaluated in this set Table 1

The criteria for selecting the designed molecules were as follows: the IC_50_ values should be less than ten micromolar, the affinity for the molecular target should be more negative than -8 kcal/mol (keeping in mind that the CBZ nucleus presents a value of -7 kcal/mol), and the logP values should fall within the range of 2 to 4, as this range has experimentally shown promising results. Finally, the toxicity profile should be equivalent to or better than that of existing drugs.

The authors have not given a detailed accounts of the design of 57 compounds using various methods. Authors must give details and elaborate on this design?

- **Response:** According to your question, in this paragraph described in the manuscript we establish the criteria for the design of the molecules.

“To explore this, 57 structural changes were made, including homologous series, bioisosteric changes, and ring replacements in the nucleophilic and electrophilic positions of the CBZ nucleus.”

This is done by seeking to optimize the subsequent synthesis process using a commercial building block such as CBZ for subsequent optimal scaling.

Is selected 6 descriptors in QSAR models, Molecular docking interaction how contribute for these 57 compound design?

- **Response:** Thank you for your question. The descriptors contribute to the pharmacophoric core of carbamazepine. Our 57 changes were made to the auxophores of carbamazepine, which kept the pharmacophoric grouping intact, ensuring biological activity but improving its pharmacophoric and dynamic attributes. It was added to the text pharmacophoric and dynamic attributes.

Authors have designed 57 compounds and selected those compound having IC50 value less that 10 micro molar. What is the logic for selecting such a large value? Generally compound with less than 1 micromolar value are rational to select?

- **Response:** Thanks for the question. If we are clear, values of 1 micromolar are ideal for rational design. However, the auxophoric changes generated predictions close to 8 micromolar, but since our only criterion is not the IC_50_ value, we consider that a synergy between the logP affinity and the activity value made the candidate promising, as was demonstrated in the in-house tests. vitro.

The authors claim that CR80 is less toxic. However, to my knowledge, compounds with statistical values above 0.5 for HERG, AMES, and RAT TOXICITY are not good. In table CR80, AMES is 0.552, and RAT toxicity is 0.601, which is quite high. The authors claim that CR80 has the best toxicity profile. However, one can see from table 2 that compounds 57, 58, 70, 73, 77, and even 79 have better profiles than CR80.

- **Response:** Thank you for your comment, we would like to clarify why the selection of CR80

The choice of the candidate did not depend exclusively on one or another criterion, our proposal is that with respect to Hit CBZ the pharmacodynamic and pharmacokinetic attributes would be improved; in this case, candidates 57, 58, 73, and 79 have log values greater than 4 lo which makes them disposable and 70 does not exceed the affinity, for that reason in this coma we do not select the mentioned candidates.

For readers' clarity, the paragraph was written as follows: While the remaining three are outside the established range, these candidates have security profiles equivalent to CBZ and commercial drugs. However, candidate 80 has the best toxicity profile, better than the three selected candidates, so it will proceed to the synthesis stage.

Authors have predicted IC50 value of compound CR80 to be 8.1 mircomolar. However, while performing invitro analysis authors used concentration only upto 1000 nanomolar? What its rationale for these dosage?

- **Response:** Thank you for the question. It clarified why the reported results are lower than those predicted by the neural network. Therefore, we allow ourselves to clarify the following in the document.

We analyzed the cytotoxic effect on the human lymphoma-derived tumor cell line U-937 and the healthy fibroblast line L-929 to assess the selectivity of the synthesized drug, the cytotoxic effect of CR80 on the U937 and L929 cell lines was evaluated at a concentration of 7.9 micromolar, as predicted by the neuronal network (refer to Table 2, entry 80). This concentration caused cytotoxicity in both the U937 and healthy L929 lines, resulting in a non-viable selectivity index (data not included). It is crucial to understand that the developed prediction function does not support forecasting the SI selectivity index, as it relies solely on the IC_50_ values of cancer cells; this represents a methodological limitation we must consider. Although CR80 showed promising activity, its low safety profile led us to conduct dilutions in the nanomolar range, concentrating on four levels: 200, 400, 800, and 1000 nM. Doxorubicin was used as a positive control at 25 nM.

We confirmed that the in vitro values predicted by the QSAR model developed with the INQA-ANN align when assessing the U -937 cell line. However, its impact on healthy cells, such as L929, shows toxicity at 10 micromolar. To improve the selectivity index (SI), we performed dilutions that yielded excellent SI results. This demonstrates that the synergistic approach combining LBDD and SBDD can lead to the identification of a promising new drug for in vivo trials. Furthermore, we are developing new QSAR models aimed at predicting both the activity against cancer cells and the selection index, which will serve as a crucial criterion for new lead compounds.

Reviewer #1: The manuscript is well written. Authors use In-silico approaches for selection and designing of molecule, Also, synthesis and in vitro experiments were performed. However, some additional detail are needed to enhance the its impact

In introduction, brief overview of the current therapies for acute lymphoblastic leukemia should be include. Also, rationale for selecting carbamazepine as a lead compound should be explain.

- **Response:** Thank you for your suggestion. Current therapies for the treatment of children and adults with ALL were included, and the manuscript was updated with ALL were included and the manuscript was updated.

The prognosis for patients with acute lymphoblastic leukemia (ALL) has greatly improved due to intensive multimodal treatment strategies, such as chemotherapy, high-dose chemotherapy with stem cell rescue, and radiation therapy when necessary [3]. The treatment of Adult Acute Lymphoblastic Leukemia (ALL) involves complex chemotherapy combinations and schedules typically seen in oncology. Two main chemotherapy regimens are currently used. The Berlin-Frankfurt-Münster protocol features an induction regimen, consolidation regimen, reintensification regimen, and maintenance therapy, primarily implemented in European adult ALL clinical trials. Alternatively, the hyper-CVAD regimen, created by MD Anderson Cancer Center researchers, consists of rotating two different intensive chemotherapy cycles [4].

3 Chang, J. H. C., Poppe, M. M., Hua, C. H., Marcus, K. J., & Esiashvili, N. (2021). Acute lymphoblastic leukemia. *Pediatric Blood & Cancer*, *68*, e28371.

4 Imai, K. (2017). Acute lymphoblastic leukemia: pathophysiology and current therapy. *[Rinsho Ketsueki] The Japanese Journal of Clinical Hematology*, *58*(5), 460-470.

Some more details on the molecular docking and software used for binding affinity predictions would strengthen the methodology section.

- **Response:** Thank you for your suggestion

The methodology was expanded in the manuscript as follows “The binding affinities of drugs that affect beta-tubulin, including their designed analogs and other frequently prescribed medications for treating ALL, were evaluated in kcal/mol utilizing the PDB 6QUS crystal structure [18]. Protein preparation followed the AutoDockTools protocol [19]. The co-crystallized ligand, paclitaxel, was removed using Samson software. After preparing the crystal, docking was conducted with the known active ligand, vincristine. Following validation of the docking, additional energies were calculated. The structures were modeled, and their energies were optimized in Avogadro [20] using the MMFF94s force field. Subsequently, 35 drugs and 58 designed analogs were docked with 6QUS. The grid box was set at 13 × 15 × 25 points with a grid spacing of 0.375 Å, centered at coordinates 2, 23, and 2. Calculations were conducted in triplicate, and the affinity energy of the pose with the lowest RMSD value was averaged for each compound. The interactions and distances were visualized using Discovery Studio Suite®.”

In discussion, how findings of studies correlate with potential mechanisms of CR80 through beta-tubulin should include.

- **Response** Thank you for your comments, in the manuscript we expand the role of ALL.

Additionally, we found 30 molecules specifically targeting beta-tubulin, a promising target because leukemic cells, like those in ALL, divide more rapidly than normal cells. This rapid division can enhance beta-tubulin expression, making these cells more vulnerable to microtubule-interfering agents, such as vincas (vincristine) and taxanes. Therefore, molecules with greater affinity for beta-tubulin will selectively target cells with accelerated division, meaning treatments with a higher affinity for beta-tubulin preferentially affect leukemic cells over healthy cells due to the latter dividing slower [X]

González-García, J. R., et al. (2019). "Targeting microtubules in cancer therapy." Cancer Treatment Reviews, 80, 101895.

Wang, X., Gigant, B., Zheng, X., & Chen, Q. (2023). Microtubule‐targeting agents for cancer treatment: Seven binding sites and three strategies. MedComm–Oncology, 2(3), e46.

Reviewer #2:

1. Please elaborate on the role of target selected i.e. beta tubulin in ALL

Response Thank you for your comments, in the manuscript we expand the role of ALL

Additionally, we found 30 molecules specifically targeting beta-tubulin, a promising target because leukemic cells, like those in ALL, divide more rapidly than normal cells. This rapid division can enhance beta-tubulin expression, making these cells more vulnerable to microtubule-interfering agents, such as vincas (vincristine) and taxanes. Therefore, molecules with greater affinity for beta-tubulin will selectively target cells with accelerated division, meaning treatments with a higher affinity for beta-tubulin preferentially affect leukemic cells over healthy cells due to the latter dividing slower [X]

González-García, J. R., et al. (2019). "Targeting microtubules in cancer therapy." Cancer Treatment Reviews, 80, 101895.

Wang, X., Gigant, B., Zheng, X., & Chen, Q. (2023). Microtubule‐targeting agents for cancer treatment: Seven binding sites and three strategies. MedComm–Oncology, 2(3), e46.

2. It is not stated anywhere how the data is recorded for in vitro test... duplicate or triplicate ?

Response: Thanks for the comment. In the manuscript, both in the experimental part and in the graphics, it was updated that the tests were in triplicate.

3.There is no mention of probability value for toxicity value output... so it not clear

Response: thanks for the comment. We allow ourselves to explain.

We analyzed the cytotoxic effect on the human lymphoma-derived tumor cell line U-937 and the healthy fibroblast line L-929 to assess the selectivity of the synthesized drug, the cytotoxic effect of CR80 on the U937 and L929 cell lines was evaluated at a concentration of 7.9 micromolar, as predicted by the neuronal network (refer to Table 2, entry 80). This concentration caused cytotoxicity in both the U937 and healthy L929 lines, resulting in a non-viable selectivity index (data not included). It is crucial to understand that the developed prediction function does not support forecasting the SI selectivity index, as it relies solely on the IC_50_ values of cancer cells; this represents a methodological limitation we must consider. Although CR80 showed promising activity, its low safety profile led us to conduct dilutions in the nanomolar range, concentrating on four levels: 200, 400, 800, and 1000 nM. Doxorubicin was used as a positive control at 25 nM

We confirmed that the in vitro values predicted by the QSAR model developed with the INQA-ANN align when assessing the U -937 cell line. However, its impact on healthy cells, such as L929, shows toxicity at 10 micromolar. To improve the selectivity index (SI), we performed dilutions that yielded excellent SI results. This demonstrates that the synergistic approach combining LBDD and SBDD can lead to the identification of a promising new drug for in vivo trials. Furthermore, we are developing new QSAR models aimed at predicting both the activity against cancer cells and the selection index, which will serve as a crucial criterion for new lead compounds.

4. Rationale behind the selection of CR80 is not satisfactory as the similar values are observed for other derivatives

**Response:** The criteria for selecting the designed molecules were as follows: the IC_50_ values should be less than ten micromolar, the affinity for the molecular target should be more negative than -8 kcal/mol (keeping in mind that the CBZ nucleus presents a value of -7 kcal/mol), and the logP values should fall within the range of 2 to 4, as this range has experimentally shown promising results. Finally, the toxicity profile should be equivalent to or better than that of existing drugs

Based on this, if any criterion such as toxicity was more promising but did not meet the three initial criteria; affinity, IC50 and log p were discarded.

James Guevara Pulido

Corresponding author

joguevara@unbosque.edu.co
