# Supplementary material for: Design, synthesis, and in vitro evaluation of a carbamazepine derivative with antitumor potential in a model of Acute Lymphoblastic Leukemia
Source: PLoS One. 2025 Apr 28;20(4):e0319415. doi: 10.1371/journal.pone.0319415 (PMC12036894; doi:10.1371/journal.pone.0319415)
Supplement: Attachment — Submitted filename: Reviewers Comments.doc [file pone.0319415.s006.doc]

**Reviewers Comments**

1. As author said in the abstract that 156 approved cancer therapies based on small molecules are there so they should give reference for this.
2. Rationale behind the selection of CR80 is not satisfactory, Authors must give an explanation
3. The IC 50 values are in nanomolar and some in millimolar, authors should explain why they are different. Authors must use only one unit for all the compounds.
4. Authors should give numbering to the structures of scheme.
5. Authors should incorporate 3D representation of the docking results.
6. Authors must compare the binding affinity of the compounds with the standard drug.
7. The word in vivo, in vitro, in silico must be in italics.
8. The graphical representation is missing.
9. Authors must give graphs of IR, NMR and Mass of CR80.
10. Authors must follow the referencing style given by journal.
