# Supplementary material for: Design, synthesis, and in vitro evaluation of a carbamazepine derivative with antitumor potential in a model of Acute Lymphoblastic Leukemia
Source: PLoS One. 2025 Apr 28;20(4):e0319415. doi: 10.1371/journal.pone.0319415 (PMC12036894; doi:10.1371/journal.pone.0319415)
Supplement: Attachment — Submitted filename: Response to reviewers R2.docx [file pone.0319415.s008.docx]

**Sapan Kamleshkumar Shah, Ph.D., M.Pharm.**

**Academic Editor**

**PLOS ONE**

Additional Editor Comments and Reviewer 4

Thank you for your comments, which enable us to present an improved version of the manuscript. Below, we provide a point-by-point response to the comments from the editor and referee 4.

The manuscript is ready to accept and accepted after some minor corrections.

1. As the author said in the abstract, 156 approved cancer therapies based on small molecules are available, so they should give a reference for this.

**Response: reference was added.**

**3. National Cancer Institute. Cancer Drugs - National Cancer Institute [Internet]. www.cancer.gov. 2012. Available from: https://www.cancer.gov/about-cancer/treatment/drugs.**

2. The rationale behind the selection of CR80 is not satisfactory; authors must give an explanation

**Response:** Thank you for your comment; we would like to clarify why the selection of CR80

**We want to clarify the reasons for selecting CR80. The candidate's selection was not solely based on one criterion. Our proposal suggests that, regarding Hit CBZ, the pharmacodynamic and pharmacokinetic attributes would see improvement. Candidates 57, 58, 73, and 79 have log values greater than 4, making them acceptable, whereas candidate 70 does not demonstrate sufficient affinity; therefore, we did not select the mentioned candidates in this instance.**

**Additionally, the criteria for choosing the designed molecules included the following: the IC50 values should be less than ten micromolar, the affinity for the molecular target must be more negative than -8 kcal/mol (considering that the CBZ nucleus has a value of -7 kcal/mol), and the logP values should be within the range of 2 to 4, as this range has been shown to yield promising experimental results. Lastly, the toxicity profile should be comparable to or better than that of existing drugs. Consequently, any candidates that had a more promising toxicity profile but did not satisfy the three primary criteria—affinity, IC50, and logP—were excluded from consideration.**

3. The IC 50 values are in nanomolar and some in millimolar. The authors should explain why they are different. They must use only one unit for all the compounds.

**Response: Thanks for the suggestion; the change of units referred to a way of representing a low concentration, but all the units and the graph have already been converted to micromolar.**

4. Authors should give numbering to the structures of the scheme.

**Response: this was corrected**

5. The words in vivo, in vitro, and silico must be in italics.

**Response: this was corrected in the manuscript.**

6. Authors must provide graphs of IR, NMR, and Mass of CR80.

**Response In the SI, IR, and MS-HR were added. NMR was already in the SI**

7. Authors should incorporate a 3D representation of the docking results.

Scheme 2. Non-covalent interactions CR80 and 3D representation

8. Authors must compare the binding affinity of the compounds with the standard drug.

**Response: The manuscript in the following paragraph is compared to the standard drug described.**

**“We used the Beta-tubulin structure with the code 6QUS as the molecular target, obtained from the RCSB Protein Data Bank (RCSB PDB). This protein is a microtubule-organizing protein that specifically binds to the minus end of non-centrosome microtubules and regulates their dynamics and organization. Vincristine was used as a validation ligand with demonstrated activity and compared with the designed analogs (entry 2 table 1)[31].”**

9. The graphical representation is missing.

**This information can be found in an additional file titled "Figures and Schemes."**

10. Authors must follow the referencing style given by the journal.

**The referencing style has been corrected.**

James Guevara Pulido

Corresponding author

joguevara@unbosque.edu.co
