# Supplementary material for: Design, synthesis, and in vitro evaluation of a carbamazepine derivative with antitumor potential in a model of Acute Lymphoblastic Leukemia
Source: PLoS One. 2025 Apr 28;20(4):e0319415. doi: 10.1371/journal.pone.0319415 (PMC12036894; doi:10.1371/journal.pone.0319415)
Supplement: Attachment — Submitted filename: reviewer comments.docx [file pone.0319415.s004.docx]

**1. Please elaborate on the role of target selected i.e. beta tubulin in ALL**

**2. It is not stated anywhere how the data is recorded for in vitro test... duplicate or triplicate ?**

**3. There is no mention of probability value for toxicity value output... so it not clear**

**4. Rationale behind the selection of CR80 is not satisfactory as the similar values are observed for other derivatives**
